# Family eczema-history in 2-year olds with eczema; a prospective, population-based study. The PACT-study, Norway

**DOI:** 10.1186/1471-5945-11-11

**Published:** 2011-05-20

**Authors:** Marit Saunes, Torbjørn Øien, Ola Storrø, Roar Johnsen

**Affiliations:** 1 Department of Public Health and General Practice, Norwegian University of Science and Technology, Trondheim, Norway; 2 Department of Dermatology, St. Olavs Hospital, Trondheim University Hospital, Norway

## Abstract

**Background:**

A maternal line of inheritance regarding eczema has been described in several studies, whereas others find associations to both a maternal as well as a paternal line of inheritance. When studying family history of eczema symptoms, cohort studies including siblings are rare. Time point for assessing family eczema-history could be of importance when studying the associations between family eczema-history and children with eczema, as parents with unaffected children may not recall mild symptoms in other siblings or their own disease history. We therefore aimed to study the associations between reported eczema in mother, father and siblings and reported eczema in index child where information on family history was collected at two different ages of index child.

**Methods:**

Parents/children participating in The Prevention of Allergy among Children in Trondheim (PACT) study were given questionnaires on reported eczema symptoms in mother, father and siblings at 6 weeks and 1 year. When index child was 2 years of age, a detailed questionnaire on different health issues with emphasize on different allergy related disorders were filled in.

**Results:**

Both maternal and paternal reports on eczema were significantly associated with eczema in index child. Reporting family eczema-history at 1 year (N = 3087), "eczema sibling only" [adjusted odds ratio (aOR) = 3.13 (2.27-4.33)] as well as all other family-groups containing siblings with eczema were strongly associated with eczema 2 years. When family eczema-history was reported at 6 weeks (N = 2657), reporting of "eczema sibling only" was not associated to reported eczema at 2 years in index child [aOR = 1.31 (0.77-2.23)].

**Conclusions:**

Having sibling(s) with eczema strengthened the associations between maternal and paternal reports on eczema with eczema in index child only when exposure was reported at 1 year. These findings indicate that results from questionnaires-based studies of family eczema-history depend on whether or not index child has yet developed eczema.

**Trial registration:**

ISRCTN: ISRCTN28090297

## Background

Atopic eczema is a complex disease caused by a mainly unknown interaction between genetic and environmental factors [1]. The genetic component of the disease has been demonstrated in twin studies [2], and several studies have emphasized the association of atopy in the mother with the development of atopic eczema in the child whereas the evidence for association to an atopic father has been somewhat weaker [3-6]. In the last years, however, other studies have concluded that the association with both paternal as well as maternal atopy is important in the development of allergic disease in the offspring [7-10] Several candidate genes linked to the development of eczema have been identified [11], but so far only mutations in the gene encoding filaggrin (FLG) have been widely replicated [12].

When family eczema-history is studied, several investigators have studied the protective effect of having a high number of siblings on the development of allergic diseases [13,14]. However, few prospective studies from the general population have addressed the association between allergy related disorders in older siblings and the child under study [4,15].

When studying diseases in the general population, self-reported questionnaires are often used. One of the flaws with such information could be recall bias concerning how well adults remember both their own childhood diseases as well as diseases in children with mild symptoms [16-18].

The aim of this prospective study was therefore to investigate the associations between reported eczema in mother, father and siblings and reported eczema in index child at 2 years of age where family eczema-history as well as exposure was collected at two different ages of the index child.

## Methods

The Prevention of Allergy among Children in Trondheim (PACT) study is a large population- based prospective study on allergy related disorders conducted in Trondheim, central Norway. Trondheim has approximately 165 000 inhabitants and 2100 deliveries pr year.

From September 2000 all pregnant women as well as children 6 weeks, 1 year, and 2 years of age visiting their general practitioner or community based midwife were consecutively invited to attend the study. Women/parents were eligible to participate if they were able to complete a questionnaire in Norwegian. Recruitment of 6 weeks old, 1 year and 2 years old children closed June 30^th ^2006. The PACT-study is described in further detail elsewhere [19].

### -Study design

Family eczema-history and exposure variables was assessed from questionnaires when the child was 6 weeks old (Q2) and 1 year old (Q3) [19]. When the child was approximately 2 years of age, the parents answered a questionnaire on the child's health (Q5). The questionnaires Q2 and Q3 included questions on different allergic diseases in parents/siblings (including family eczema-history) as well as on indoor climate, infections, medication, vaccines, day-care, exposure to nicotine, pets, breastfeeding and diet. Q5 included questions on different health issues in index child with emphasize on allergy related disorders. The latter questions were adopted from the International Study of Asthma and Allergies in Childhood (ISAAC) protocol and modified to fit the actual age group [20].

By March 2009 some 2657 parents (54.6%) had completed Q2 as well as the child's health-questionnaire at the age of 2 years (Q5), and these are included in the 6 weeks cohort. The 1 year cohort comprises 3087 parents (60%) who had completed Q3 together with Q5 (table 1).

**Table 1:** Characteristics of the two study populations with exposure reported at 6 weeks and 1 year.

		Exposure reported at 6 weeks, N = 2657			Exposure reported at 1 year, N = 3087		
***Reported exposure ***^***a***^		**n**	**%**	**(95% CI)**	**n**	**%**	**95% CI**

Gender	Male	1333	50.2	(48.3-52.1)	1529	49.5	(47.8-51.3)
Homeowner	Yes	2242	84.4	(82.9-85.7)	2680	86.8	(85.6-88.0)
Cat	Yes	239	9.0	(7.9-10.1)	266	8.6	(7.7-9.7)
Dog	Yes	247	9.3	(8.2-10.5)	249	8.1	(7.1-9.1)
Bird	Yes	63	2.4	(1.8-3.0)	70	2.3	(1.8-2.9)
Dampness	Yes	105	4.0	(3.2-4.8)	117	3.8	(3.1-4.5)
Maternal current smoking	Yes	189	7.1	(6.2-8.2)	453	14.7	(13.4-16.0)
Paternal current smoking	Yes	399	15.0	(13.7-16.4)	499	16.2	(14.9-17.5)
Premature birth, < 37 weeks	Yes	75	2.8	(2.2-3.5)	75	2.4	(1.9-3.0)
Birth-weight	<2500gr	74	2.8	(2.2-3.5)	105	3.4	(2.8-4.1)
	2500-3999gr	1937	72.9	(71.2-74.6)	2196	71.1	(69.5-72.7)
	≥4000gr	623	23.4	(21.8-25.1)	691	22.4	(20.9-23.9)
Breastfeeding now	Yes	2481	93.4	(92.4-94.3)	1114	36.1	(34.4-37.8)
Number of siblings	0	1172	44.1	(42.2-46.0)	1343	43.5	(41.7-45.3)
	1	920	34.6	(32.8-36.5)	1033	33.5	(31.8-35.2)
	2 or more	533	20.1	(18.6-21.6)	711	23.0	(21.6-24.6)
Any kind of infection, child	Yes	903	34.0	(32.2-35.8)	2983	96.6	(95.9-97.2)
Antibiotics ever, child	Yes	58	2.2	(1.7-2.8)	706	22.9	(21.4-24.4)
							
	**mean**	**range**	**SD**		**mean**	**range**	**SD**
Maternal age	30.1	17-48	4.49		29.98	17-48	4.46

^a ^Number of missing varies

These two cohorts (6 weeks, N = 2657 and 1 year, N = 3087) were used to prospectively study reported family eczema-history and association to eczema in index child reported at 2 year.

### -Study variables

*-The outcome variable *studied was ever eczema reported at the age of 2 years (Q5). The index child was defined as having eczema if both questions "Has your child *ever *had eczema?" and "Has your child *ever *had an itchy rash which was coming and going for at least 6 months?" were answered positively [21].

Family eczema-history and exposure variables were assessed in Q2 and Q3.

#### -Family eczema- history

Eczema in mother, father or sibling was defined as "yes" if any one of the questions "have you, the child's father or any of your joint children ever had eczema" or "have you, the child's father or any of your joint children had eczema or used medication against eczema during the last 12 months" were ticked "yes" for mother, father or sibling.

The reporting of family eczema history was then categorized into the seven different family eczema groups possible; "mother only", "father only", "sibling only", "mother and father", "mother and sibling", "father and sibling", "mother, father and sibling".

#### -Exposure variables

The term "breastfeeding now" was used regardless of whether or not solid food or formulas was given to the child in addition to breast milk.

"Current smoking mother" was regarded as "yes" when the question "do you smoke now?" was answered positively.

Maternal and paternal level of education was not accounted for in the original questionnaire. Thus, homeowner status from the questionnaire was used as a proxy for socioeconomic status.

"Dampness" was defined "yes" when the sum of 8 different questions concerning damp/mould at home was 3 or more.

### -Ethics

All parents signed a written consent form to participate in the PACT study. The study was approved by the Regional Committee for Medical Research Ethics and the Norwegian data Inspectorate Board (Ref 120-2000) (Ref 2003/953-3 KBE/-).

### -Statistics

Univariate associations between eczema in index child and different family eczema-groups as well as the different exposure variables were analysed using simple logistic regression.

Associations adjusted for potential confounders were obtained by logistic regression models. Maternal age was used as a linear variable in the regression analysis. All other explanatory variables were either dichotomized or categorized. Dummy variables were made when the independent variables contained more than two categories. Family eczema-groups were also categorized, and those with no family history of eczema (mother, father or sibling) were set as reference group.

The logistic regression models were adjusted for confounding factors identified by *a priori *knowledge to the problem being studied. In the final multivariable regression analysis, adjustments were made for gender, homeowner, current smoking mother, breastfeeding, keeping a dog and age of mother at the time of birth.

A separate model was made for older siblings with and without eczema. In addition to adjustment for the *a priori *defined confounding factors, adjustment was made for eczema mother and eczema father. Interaction between eczema mother and eczema father was tested.

In the multivariable analysis the number of missing varied for each variable under study, but never exceeded 9%.

Unadjusted and adjusted associations are presented as odds ratios (OR, aOR). 95% confidence intervals (CI) were estimated for binominal distributed data.

Statistical Package for Social Science version 15.0 (SPSS inc., Chicago; IL, USA.) and STATA version 11.2 for Windows (STATA Corporation, College Station, TX, USA) were used for the analyses.

## Results

Some 13.7% and 14.2% of the girls, and 15.9% and 16.1% of the boys reported eczema at 2 years of age in the 6-week cohort (N = 2657) and in the 1-year cohort (N = 3087), respectively.

When exposure was reported at 6 weeks, only some 6% of the index children with eczema had had their first symptoms of eczema, whereas the corresponding number was about 80% among those who reported exposure at 1 year.

Some 77% of children in the 6-week cohort also attended the 1-year cohort. Between 6 weeks and 1 year, some 9.6% of these parents changed their answer from "no" to "yes" regarding whether or not the index child had a sibling with eczema or not. Among those with eczema in index child reported at 2 years, some 28.5% changed their answer from "no" to "yes" from 6 weeks to 1 year, as opposed to some 6.4% among those without eczema in index child 2 years (data not shown).

In univariate analyses, keeping a dog was the only statistically significant environmental factor associated with reported eczema at 2 years of age in both cohorts.

When reporting family history at 6 weeks, the association between eczema in index child at 2 years and different eczema- groups containing only one or both of the parents, both "eczema mother only" and eczema father only" were statistically significant in the univariate analyses [(OR, 1.61; 95% CI 1.18-2.21), (OR, 1.69; 95% CI 1.16-2.46)] (table 2). When adding reporting of eczema in a sibling to eczema in one or both of the parents, we found "eczema mother and sibling, not father" and "eczema mother, father and sibling" to be significantly associated with eczema in the index child at 2 years [(OR, 3.41; 95% CI 1.91-6.10) and (OR, 3.15; 95% CI 1.42-7.02)].

**Table 2:** Univariate association between different family eczema-groups and eczema 2 years.

	Proportion of children with eczema 2 years	OR (95% CI)		Proportion of children with eczema 2 years	OR (95% CI)
**Reported 6 weeks**			**Reported at 1 year**		
No family eczema ^a^	12.5% (217/1738)	1.0	No family eczema ^a^	10.7% (201/1885)	1.0
Eczema mother only	18.7% (61/326)	1.61 (1.18-2.21)	Eczema mother only	16.7% (59/354)	1.68 (1.22-2.30)
Eczema father only	19.4% (39/201)	1.69 (1.16-2.46)	Eczema father only	18.1% (39/215)	1.86 (1.27-2.71)
Eczema mother and father, not sibling(s)	19.7% (13/66)	1.72 (0.92-3.21)	Eczema mother and father, not sibling(s)	16.0% (12/75)	1.60 (0.85-3.01)
Eczema sibling(s) only	16.9% (22/130)	1.43 (0.88-2.31)	Eczema sibling(s) only	25.5% (67/263)	2.86 (2.09-3.92)
Eczema mother and sibling(s), not father	24.1% (19/79)	3.41 (1.91-6.10)	Eczema mother and sibling(s), not father	33.9% (42/124)	4.29 (2.88-6.40)
Eczema father and sibling(s), not mother	23.1% (9/39)	2.10 (0.99-4.49)	Eczema father and sibling(s), not mother	37.5% (27/72)	5.03 (3.05-8.28)
Eczema mother, father and sibling(s)	31.0% (9/29)	3.15 (1.42-7.02)	Eczema mother, father and sibling(s)	40.0% (14/35)	5.59 (2.80-11.16)

^a ^No family eczema is the reference point for all other family eczema-groups

Corresponding univariate analyses of different family history eczema-groups reported at 1-year showed no differences in associations with eczema in index child at 2 years for any of the groups containing one or both of the parents without a sibling compared to 6 weeks. For all groups including eczema in siblings there were a consistent and highly significantly association with eczema at 2 year in the index child (table 2).

In the adjusted model, we found a significant association between "eczema mother only" and "eczema father only" reported at 6 weeks and eczema in the index child at 2 years [(aOR, 1.57; 95% CI 1.13-2.18), (aOR, 1.73; 95% CI 1.18-2.55)] (table 3). No association was found with "eczema sibling only" (aOR, 1.31; 95% CI 0.77-2.23).

**Table 3:** Adjusted association between different family eczema-groups and eczema 2 years ^a,b^.

	Reported at 6 weeks		Reported at 1 year	
	**aOR**	**(95% CI)**	**aOR**	**(95% CI)**
Eczema mother only	1.57	(1.14-2.18)	1.62	(1.17-2.24)
Eczema father only	1.73	(1.18-2.55)	1.98	(1.35-2.90)
Eczema mother and father, not sibling	1.83	(0.97-3.42)	1.73	(0.91-3.28)
Eczema sibling only	1.31	(0.77-2.23)	3.13	(2.27-4.33)
Eczema mother and sibling, not father	2.59	(1.34-5.00)	4.48	(2.96-6.78)
Eczema father and sibling, not mother	2.14	(0.99-4.63)	5.43	(3.27-9.02)
Eczema mother, father and sibling	3.78	(1.66-8.63)	6.25	(3.09-12.65)

^a ^) No family eczema is the reference point for all other family eczema-groups

^b ^) Model adjusted for gender, homeowner, current smoking mother, breastfeeding, keeping a dog and age of mother at time of birth.

When adapting the same model on reported eczema in mother, father and sibling at 1 year, we found a significant association with eczema at 2 years in all groups but "eczema mother and father" (Table 3). As opposed to the family eczema-history reported at 6 weeks, a significant association was observed for "eczema sibling only" (aOR, 3.13; 95% CI 2.27-4.33). Adding eczema in sibling to either of the eczema parent groups significantly strengthened the associations in the 1 year cohort; "eczema mother and sibling, not father" (aOR, 4.48; 95% CI 2.96-6.78), "eczema father and sibling, not mother" (aOR, 5.43; 95% CI 3.27-9.02).

In the model testing association between siblings with and without eczema and association to eczema in index child 2 years, we found a significant association for siblings with eczema in the 1 year cohort only (aOR, 2.18; 95% CI 1.63-2.92). No interaction between eczema mother and father was found in either of the cohorts (*p *for interaction = 0.48 at 6 weeks, *p *for interaction = 0.10 at 1 year) (data not shown).

## Discussion

In this large population based study with reports of family eczema-history at two different points of time, we found that having either parent with eczema was significantly associated with reported eczema at the age of 2 years. The associations were consistent when reported at 6-weeks as well as 1 year. Having one or several siblings with eczema, with or without either of the parents with eczema reported at 1 year, was also strongly associated with reported eczema at 2 years and significantly stronger for siblings only.

We have data on exposure reported at 6 weeks and 1 year. Although the reporting of exposure was at two different points of time, the two cohorts are comparable. Some 77% of those who answered Q2 (6 weeks) also answered Q3 (1 year) as well as Q5 (2 years). The two cohorts are generated from the same geographic area (the city of Trondheim), during the same time-period, and by the same midwifes and GPs.

Although eczema is a relatively prevalent disease among children in the western world, most children have mild degree of disease, making the diagnosis and differentiating between different phenotypes of the disease as well as mild cases challenging [21-23]. We found the prevalence of reported eczema 2 years to about 15% both among those who reported the family eczema history at 6-week and those who reported at 1 year, indicating a high reliability of the questions. Any misclassification of eczema cases might therefore be non-differential, and if so may have diluted the associations.

Several studies describe a maternal line of inheritance concerning eczema [5,6,22]. This maternal line of inheritance has led investigators to hypothesize that environmental influences operating *in utero *or in early infancy may be essential in determining disease expression [24]. In this study eczema in the index child was significantly associated with eczema in mothers as well as fathers. This finding is in accordance with several others [8,9,25] and not supportive to the hypothesis of paternal genomic imprinting. However, parental recall bias should be taken into consideration also here. Any one parent who followed the child was asked to participate in the PACT-study. One limitation in this study is the fact that we don't know which parent filled in the questionnaire. Since many of the women were included during pregnancy and during their child's first year of living, we assume that mothers most likely have accepted to participate in the study when visiting her GP or midwife. It is therefore most likely that mothers have filled in the questionnaires. In both cohorts more boys than girls reported eczema at age 2 years. Despite this, more mothers than fathers reported to have ever eczema. When studying recall bias in parental questioning, a German group found that mothers tended to report more atopic diseases in a second questionnaire than in the first, whilst fathers were influenced by their child's development of atopic disease [16]. In families without childhood eczema the sensitivity for mothers reporting paternal eczema was lower than in families with at least one child with eczema. The specificity was about the same [26]. Although effect size is small in a population setting, filaggrin haploinsufficiency is a highly penetrant trait, and associated with increased eczema severity [27]. It is therefore likely that severe eczema in any one of the parents represent a greater risk of eczema in the offspring. It seems also likely, that mothers more often would report a positive paternal history of eczema if the disease was severe or persisted into adulthood. The latter is supported by a Swedish study, who found that recall of childhood eczema history among adults was influenced by several factors such as high prevalence of eczema after the age of 15, more visits to the physician after the age of 15, more hand eczema and more sick-leave due to eczema [17]. Since mothers most likely have filled in our questionnaires, an overestimation of the association between paternal eczema and eczema in index child is possible. This might be due either to more severe eczema in fathers or increased awareness due to development of eczema in one of the children. Both could explain the lack of a maternal line of inheritance in this study.

When family history of eczema is studied, allergy related disorders in siblings are seldom accounted for in the risk analysis. We found that, although having any one parent with eczema was associated with eczema in index child at 2 years, having one or several siblings with eczema together with mother and/or father with eczema was strongly associated with eczema in index child 2 years when reports were collected at 1 year. This association was also seen when eczema was reported only in sibling(s). There are different interpretations of these findings. One possible explanation could be that this is a reflection of a parental genetic disposition with incomplete penetrance [4]. Mutations in the gene encoding filaggrin (FLG) have been identified as a strong predisposing factors for eczema [28] and especially severe phenotypes of the disease [29], but other candidate genes are also under investigation [11]. Different environmental factors can alter the expression of different genes, as have been showed with the exposure to cat within the first year of life in those who carry mutations of FLG [30]. Other environmental factors such as early colonisation from maternal microbial flora as well as shared environment among siblings with the same genetic predisposition may act in a similar way and explain the "eczema-sibling-effect" in this study.

The "eczema-sibling-effect" was not significant when reported at 6 weeks. Awareness of disease in first child with mild disease might be absent until same kinds of symptoms as e.g. dry skin is observed in second child. Also, in mild cases of eczema a significant proportion of the children are disease free by the age of 3 years [31]. In this study, some 28.5% of parents with eczema in index child at 2 years changed their answer from "no" when reported at 6 weeks to "yes" when reported at 1 year regarding the question on whether or not siblings had eczema, as opposed to 6.4% among those without eczema in index child. These findings could be interpreted as an increase in awareness since some 80% of index children had developed symptoms on eczema at age 1 year. Another interpretation is that some of the older siblings have not yet been diagnosed with eczema. This is however less likely, since a majority of children with eczema starts with their disease during their first year of life [31].

In a German study of children 9-11 years old having two first degree relatives with the same atopic disease was highly associated with eczema [4]. Diepgen & Blettner found a stronger correlation between siblings than between siblings and parents for all atopic diseases, also atopic dermatitis [5]. Eczema in an older sibling was also found to be an independent risk factor for eczema among 4-years old in Sweden [9].

When only some 60% of those finishing the questionnaires on exposure were answering the questionnaires on health, one could argue that the data are prone to self-selection. In both cohorts the lost-to-follow-up group contained more current smoking mothers and fewer homeowners, indicating a lower socio-economic status (data not shown). However, other exposure data, including reported family eczema-history did not differ among those who followed up and those who did not.

Another limitation of the study was the reporting eczema in parents and siblings without a clinical verification. The question "have you, the child's father or any of your joint children ever had eczema" is a core ISAAC questions, but is most often used in combination with a question on rash located in typical places or diagnosis verified by a doctor. To the best of our knowledge, the question has not been validated in an adult population. However, the almost similar phrasing "have you ever had childhood eczema" was validated among adults in a Swedish population. The sensitivity and specificity of this question was 89.9% and 70.7%, respectively [32], and an overestimation of the reported prevalence of childhood eczema among adults is therefore likely. Regarding the use of this question among children (siblings), we have validated this in a former publication [21]. This question alone, without a question identifying rash on typical location, gave a sensitivity of 96.8% and a specificity of 68.0% when validated against the UK Working Party Criteria. Adding a question of rash on typical locations decreased the sensitivity whereas the specificity increased. This gives reason to believe, that there might be an overestimation of reported eczema among the siblings, since other forms of dermatitis in young children, such as seborrhoeic dermatitis as well as nappy dermatitis might have been included. However, in case of such a misclassification, there is no reason to believe that this has changed from 6 weeks to 1 year, and could therefore not explain the differences in reporting of eczema in siblings in the two cohorts.

The strengths of this study are the large number of unselected participants as well as the prospective design. The consistency of reported eczema indicates a high reliability of the questions and the prevalence of reported eczema is well in line with the prevalence found in the PACT endpoint-study [21]. In addition, the focus is on eczema-groups only, since other studies have showed that parental eczema may be a better marker for eczema in the offspring than other parental atopic diseases [8,33].

## Conclusions

We found that reporting having a sibling with eczema at 1 year was significantly associated with reported eczema at 2 years. Eczema in mother as well as eczema in father was both associated with eczema 2 years. When family eczema-history was reported when index child was 1 years of age, the associations with eczema 2 years were significantly stronger for both parents if sibling(s) of the index child had eczema, and association was significantly stronger for sibling(s) reported at 1 year compared to 6 weeks. However, although siblings had eczema, the shared environment by mother and child did not result in any difference between maternal and paternal associations to eczema 2 years in index child.

The finding of different associations when family-eczema history was reported at 6 weeks compared to when family eczema-history was reported at 1 year indicate bias in information gathering and has important implications on comparability of studies measuring the effect of older sibling disease on index child`s risk of eczema.

## Competing interests

The authors declare that they have no competing interests.

## Authors' contributions

MS participated in the design of the study, performed statistical analysis and drafted the manuscript. TØ, OS and RJ conceived the study, participated in its design and co-ordination and helped draft the manuscript.

All authors read and approved the final manuscript.

## Pre-publication history

The pre-publication history for this paper can be accessed here:

http://www.biomedcentral.com/1471-5945/11/11/prepub
